# DEMENTIA FRIENDS IN INCARCERATED SETTINGS: RESULTS OF A STAFF E-LEARNING SESSION

**DOI:** 10.1093/geroni/igae098.4157

**Published:** 2024-12-31

**Authors:** Jessica Bibbo, Sarah Nicolay, Bonnie Burman, Marty Williman, Elizabeth Kinzig, Salli Bollin

**Affiliations:** Benjamin Rose, Cleveland, Ohio, United States; Benjamin Rose, Cleveland, Ohio, United States; Ohio Council for Cognitive Health, New Albany, Ohio, United States; Ohio Council for Cognitive Health, New Albany, Ohio, United States; Ohio Council for Cognitive Health, New Albany, Ohio, United States; Ohio Council for Cognitive Health, New Albany, Ohio, United States

## Abstract

Individuals aging in incarcerated settings such as prison develop dementia at a higher rate than those outside of such settings. The percentage of inmates aged 60 or older is rapidly increasing, yet there is little training for staff about dementia. The Ohio Council for Cognitive Health worked with the Ohio Department of Rehabilitation and Correction (ODRC) to develop Dementia Friends for Incarcerated Settings, an e-learning course made available to all ODRC staff. These results are from the optional and anonymous pre- and post-surveys embedded in the session; 125 of the 364 respondents completed both (Mage=53.2, SDage=9.1, Female=53.6%; Full time=95.2%). A series of McNemar Tests indicated significant improvement in one of the four true/false items measuring knowledge about dementia (Changing the environment will make no difference to a person who has dementia [False]), p <.001. A series of Wilcoxon Signed Rank Tests indicated significant improvement in five of the sliding-scale items (range: 0 indicating no agreement to 100 indicating total agreement) measuring attitudes about dementia (e.g., I am confident interacting with people living with dementia; It is possible to improve the lives of people living with dementia) (p ≤.010). Most respondents agreed or strongly agreed that the material covered in the session would be useful in their work (90.2%), that they learned new information (88.6%), and that they would recommend the session to coworkers (89.5%). These results indicate the potential impact of and underscore the need for dementia education within incarcerated settings.

